# Viral Diagnosis in Psittacine Birds: A Scientometric and Systematic Review of 47 Years

**DOI:** 10.3390/ani14111546

**Published:** 2024-05-23

**Authors:** Edma Santos Antonio, Ricardo Evangelista Fraga, Janisete Gomes Silva

**Affiliations:** 1 Programa de Pós-Graduação em Genética e Biologia Molecular, Departamento de Ciências Biológicas, Universidade Estadual de Santa Cruz, Campus Soane Nazaré de Andrade, Ilhéus 45662-900, BA, Brazil; jgs10@uol.com.br; 2 Instituto Multidisciplinar em Saúde, Universidade Federal da Bahia, Campus Anísio Teixeira, Vitória da Conquista 45029-094, BA, Brazil; ricardoefraga@hotmail.com

**Keywords:** scientometry, virus, infectious diseases, Psittaciformes

## Abstract

**Simple Summary:**

In this review, we present the differences between viral diagnostic studies in psittacines and their progress. The aim of this study was to analyze the evolution of published studies on viruses in psittacines up to 2022. We found that: (i) on average, 2.5 articles per year on viral diagnosis in psittacines have been published since 1975; (ii) the most productive research groups are concentrated in Australia, the United States, and Germany; (iii) the most important virus for psittacines is Circovirus; (iv) the diagnostic method of choice is polymerase chain reaction (PCR); and (v) the most studied psittacines belong to the Psittacidae family kept in captivity. This study provides information to guide future work on viruses in psittacines and encourages broad viral research in wild and domestic animals to prevent future outbreaks and the emergence of new viral diseases.

**Abstract:**

The first reports of viruses in psittacine birds date back to the early 1970s. Here, we elucidate the differences among these previous studies and the advances achieved. The objective of this study was to carry out a comprehensive review using both scientometric and systematic methods to analyze the evolution of published studies on viruses in psittacine birds up to 2022. The search descriptors “virus”, “diagnosis”, and “Psittaciformes” were used to find the articles of interest for this study. A total of 118 articles were manually selected, and the scientometric data were organized using the software VOSviewer^®^ version 1.6.18. The present review revealed that: (i) on average, 2.5 articles/year on the diagnosis of viral infection in psittacine birds were published since 1975; (ii) the most productive research groups are concentrated in three countries: Australia, the United States, and Germany; (iii) the most important virus in psittacine birds is the Circovirus, which causes psittacine beak and feather disease; (iv) the diagnostic method of choice is polymerase chain reaction (PCR); and (v) the most studied psittacine birds were those in the Psittacidae family that were kept in captivity.

## 1. Introduction

The order Psittaciformes (421 species) is divided into six families: Psittaculidae (Asian parrots, Australian parrots, and lories), Psittacidae (New World and African parrots), Psittrichasidae (Indian Ocean *Coracopsis* and New Guinean *Psittrichas*), Cacatuidae (distributed across Indonesia, Australia, Papua New Guinea, the Philippines, and the Solomon Islands), Strigopidae, and Nestoridae (both endemic to New Zealand) [1,2].

Psittaciformes, with 163 species, ranks second among all bird orders in the number of vulnerable or threatened bird species [1]. Illicit wild bird traffic to supply wild pet markets is the major threat to the conservation of these species [3]. The confinement of a wild bird can lead to its inability to perform species-characteristic behaviors and may result in abnormal behaviors and compromised immunity [4,5,6]. It has already been demonstrated that captive birds have a lower capacity to respond to antigens and may, therefore, be more susceptible to infections by pathogens such as viruses [6]. 

Viral investigations in psittacine birds are not new. The first reports of viruses in psittacine birds date back to the early 1970s, when the presence of the pox virus in *Agapornis* was detected by German researchers using electron microscopy [7,8]. Since then, several other viruses have been detected in psittacine birds: adenovirus, *Bornavirus*, *Circovirus*, coronavirus, endornavirus, *Flavivirus*, herpesvirus, *Influenza*, papillomavirus, paramyxovirus, *Picobirnavirus*, picornavirus, polyomavirus, poxvirus, and reovirus [9,10,11,12,13,14,15,16]. Therefore, it is paramount to understand the methodological differences among the several studies and the advances achieved. Scientometry, a sub-discipline of sociology and information sciences, measures scientific production in a given area based on bibliometric indicators [17]. 

Few scientometric or bibliometric studies have been carried out on bird viruses [18,19,20,21,22,23,24], and none have been conducted on psittacine birds. The majority of reviews conducted so far have focused on avian influenza. Afanador-Villamizar et al. [18] assessed 16 years of serological and molecular studies on avian influenza in Latin America. Liverani et al. [19] conducted a bibliometric review of studies on influenza in birds in Southeast Asia. Rathinasabapathy and Kopperundevi [20], as well as Selvaraj [21], evaluated all global studies on avian influenza, with the former covering the period from 1971 to 2014 and the latter from 1961 to 2016. Both reviews aimed, among other things, to identify the countries, authors, and journals with the highest production, as well as the linguistic distribution of studies. In 1988, King [22] conducted a review of all scientific production on avian virology between 1976 and 1986. Zaib et al. [23] measured research on the avian leukosis virus, focusing on co-author network analysis. Silva et al. [24] conducted, for the first time, a scientometric review of viruses focusing on a specific group of birds, quantifying all research on Circovirus in pigeons until the year 2022. 

The present review aims to provide information to guide the choice of diagnostic method and types of viruses for future investigations in psittacine birds in each region around the world, as well as to promote new studies in avian virology to fill in these knowledge gaps. To that end, the objective of this study was to carry out a scientometric and systematic review to analyze the evolution of published studies on viral diagnosis in psittacine birds from 1975 up to 2022.

## 2. Scientometric and Systematic Survey

### 2.1. Survey Design 

The search for indexed scientific publications was carried out using four databases: Dimensions^®^, PubMed^®^, Scopus^®^, and Web of Science^®^, all compatible with VOSviewer^®^ version 1.6.18, a software that can be used to build and visualize scientometric networks [25]. 

The search descriptors used to find the articles of interest for this study are listed in Table 1. Two exclusion criteria were used for the selection of articles from the databases: (i) articles published from 2023 on and (ii) research articles that did not report viral diagnosis in psittacine birds. 

For the analysis of the production and map construction, we used indicators of co-authorship (Co-authorship versus Authors), organizations (Co-authorship versus Organizations), and country (Co-authorship versus Countries). For the analysis regarding authors, the parameters used were a maximum limit of ‘25’ authors per article and a minimum of ‘2’ articles per author. Regarding countries, the parameters used were a maximum limit of ‘25’ countries per article and a minimum of ‘1’ article per country. For the analysis regarding organizations, the parameters used were a maximum limit of ‘25’ organizations per article, a minimum of ‘1’ article per organization, and ‘4’ citations per organization. 

In addition to the scientometric data, the following information was recovered from reading the articles: (i) year of publication of the article, (ii) country of origin of the birds analyzed, (iii) kinds of viruses identified, (iv) diagnostic methods used, (v) classification at the family level of the birds studied, and (vi) status of bird allocation (captivity/wild). 

### 2.2. Article Selection 

VOSviewer^®^ version 1.6.18 creates maps using bibliographical data from a single database. Therefore, we evaluated the articles from the four aforementioned databases following the established criteria and chose the database with the largest number of selected articles to be imported into the software. The database with the largest number of selected articles was Dimensions^®^, followed by PubMed^®^, Scopus^®^, and Web of Science^®^ (Table 2). The same distribution pattern was seen after the evaluation and exclusion. On average, 27.75% (± 0.73%) of the articles found in each database did not fit into the criteria to be chosen. 

Out of the 135 scientific articles recovered using Dimensions^®^, 97 were selected. This database was chosen for the analysis using VOSviewer^®^ version 1.6.18. In addition to the 97 selected articles for the scientometric analysis, a total of 21 articles recovered and selected from the other three databases were included in the systematic review (Table 2), totaling 118 articles.

## 3. Temporal Distribution: 47 Years of Research

**Figure 1:**
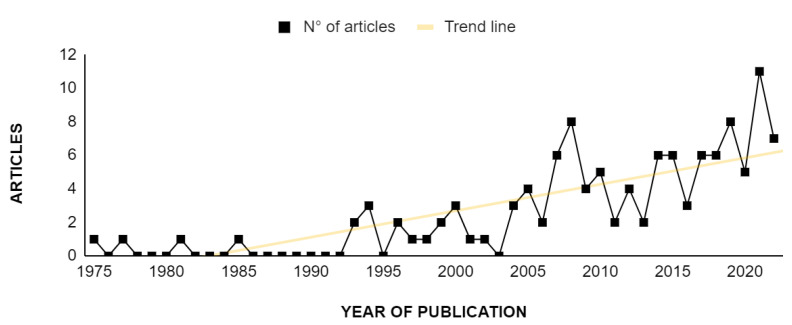
Temporal distribution of research articles on viral diagnosis in psittacid birds published from 1975 to 2022 and indexed in the databases Dimensions^®^, PubMed^®^, Scopus^®^, and Web of Science^®^.

In the historical series, the scientometric review on viral diagnosis in psittacid birds revealed an average of 2.5 articles per year over the last 47 years (from 1975 to 2022), ranging from 0 to 12 articles per year (Figure 1). 

The temporal distribution of articles was low between 1975 and 1992, ranging from 0 to 1 article/year. From 1993 to 2006, there was an increase, reaching a still low average of 1.8 articles/year. From 2007 on, there was an increase in the number of articles on this theme, with an average of 5.1 articles/year (2007–2020), ranging from 2 to 8 articles/year. For the year 2021, a total of 11 articles was registered, which was considered an expressive increase in the number of publications not repeated in the following year (2022), when the number of publications went back to the pattern observed between 2007 and 2020 (7 articles).

In order to understand the expressive increase in publication on the theme in 2021, it is worthwhile to remember the latest and one of the most important developments in the area of virology. At the end of 2019, a new coronavirus affecting humans was detected: SARS-CoV-2 [26]. COVID-19, the disease caused by this virus, was disseminated worldwide during 2020, leading to a pandemic of the disease that lasted until 2021. It is likely that COVID-19 has brought attention to studies of viruses not only in human virology but also on studies in other animals due to research pointing out the zoonotic origin of the disease and the link to wild animals [27]. Furthermore, during the COVID-19 pandemic, the world population was advised to maintain social isolation and/or work remotely [28,29]. Thus, universities had periods of no presential classes [30], and in this scenario, researchers probably had more time to devote to writing and finalizing manuscripts for publication.

## 4. The Largest Research Groups on Viruses in Psittacine Birds

### 4.1. Authors

**Figure 2:**
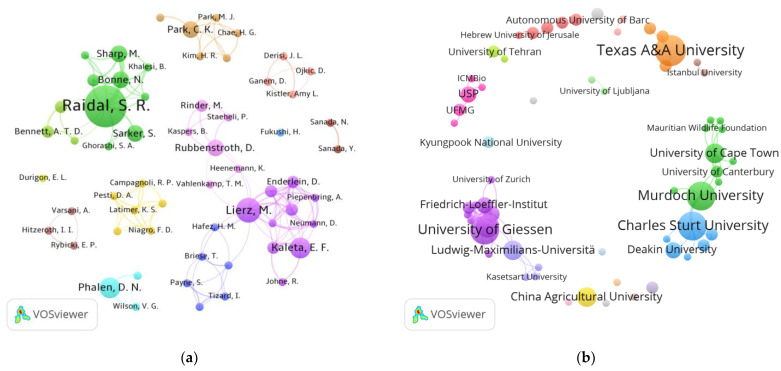
Scientometric network of the (**a**) authors and (**b**) organizations that published the most articles on viral diagnosis in parrots between 1975 and 2022 and had the articles indexed in the Dimensions^®^ database. The different colors of the clusters, formed by authors and institutions, indicate research conducted in partnership among its members. Images generated by VOSViewer^®^ version 1.6.18 software.

A total of 482 authors were identified in the 97 articles selected from Dimensions^®^, an average of 5 authors/publication. The ten most productive researchers on viral diagnosis in psittacine birds were Raidal S.R. (10/97; 10.3%), Lierz M. (6/97; 6.2%), Kaleta E.F. (5/97; 5.1%), Phalen D.N. (5/97; 5.1%), and Bonne N. (4/97; 4.1%) (Figure 2a; Table 3).

Raidal, S.R., the most productive author in the area of viral diagnosis in psittacine birds (Figure 2a: green), is a professor at the School of Agricultural, Environmental, and Veterinary Sciences at the Charles Sturt University, a public Australian university, and his studies are focused on infection by circoviruses [31]. 

Despite Raidal being the most productive researcher, the largest production network was formed by Lierz, M. (Figure 2a: lilac cluster), the author with the highest number of connections linked to at least two clusters (dark blue and pink). The joint clusters linked to Lierz represent the largest research groups in this area in Germany. Lierz is a veterinarian whose specialty is birds and a researcher at Justus-Liebig-University Giessen, a public German university [32]. 

Another researcher who stands out in the scientific production map is Phalen, D.N. Phalen is a veterinarian and a professor at The University of Sydney, a public Australian university [33]. Among his selected articles, there was no partnership with other high-impact Australian authors; thus, no link with clusters of other Australian authors was formed. 

### 4.2. Organizations and Countries

A total of 79 organizations were involved in the 97 articles selected from Dimensions^®^ (Figure 2b). The 15 most productive institutions on viral diagnosis in psittacine birds are listed in Table 4.

**Figure 3:**
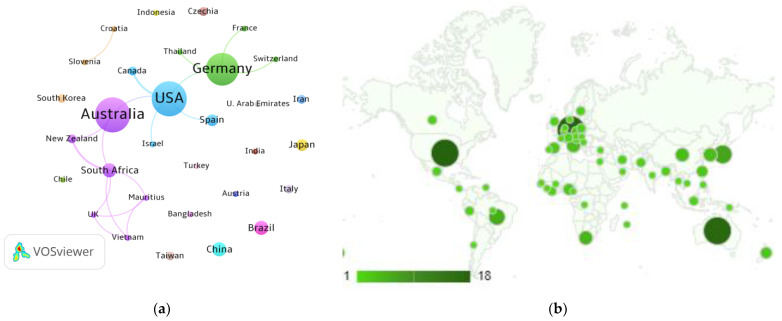
(**a**) Scientometric network of countries of origin of studies on the diagnosis of viruses in parrots between 1975 and 2022 that had their articles indexed in the Dimensions^®^ database, image generated by VOSViewer^®^ version 1.6.18. The different colors of the clusters indicate the countries most closely related in terms of conducting research on the topic addressed here. (**b**) Origin of parrots submitted to virus diagnosis according to the number of articles published between 1975 and 2022 and indexed in the Dimensions^®^, PubMed^®^, Scopus^®^, and Web of Science^®^ databases.

The selected articles originated from 31 countries. Most studies were carried out by researchers in the United States (17/97), Australia (17/97), and Germany (15/97), followed by South Africa, Brazil, and China, each with four articles published (Table 3; Figure 3a). Although the studies were carried out in 31 countries, the origin of the psittacine birds studied is much more dispersed. The birds originated from at least 55 different countries (Figure 3b), among which Germany was cited in 18 articles, the United States was cited in 18 articles, and Australia was cited in 17 articles, followed by Japan (n = 7), Brazil (n = 6), South Africa (n = 5), China (n = 4), South Korea (n = 4), and Italy (n = 4). The other 46 countries showed up in 1 to 3 articles each.

The original geographical distribution of most psittacine birds is South America, Oceania, and South Asia, and the key countries in each continent are Brazil, Australia, and Indonesia, respectively [1]. Six studies were found on psittacine birds originating from Brazil and two from Indonesia. Even though Australia has a lower diversity of psittacine birds than the two aforementioned countries, it was the place of origin for birds in 17 articles. 

Most of the more relevant authors on this theme are professors at public universities. The Australian government sees public universities as a good source of economic, technological, and social development. An Australian study compiled data that indicated a direct relationship between research carried out at universities and economic prosperity since the countries that invest the most in research at universities tend to have a higher GDP per capita. In this respect, Australia is among the countries that invest the most in higher education [34]. This might be a likely explanation why Australian authors were found among the most productive worldwide regarding studies of viruses in psittacine birds, whereas Brazil and Indonesia probably do not have sufficient investment to stand out in this area. 

Germany and the United States also ranked at the top in countries and organizations of origin of the studies of the birds. In contrast to Australia, they are not countries with a high natural diversity of psittacine birds. Chan et al. [35] indicated that the United States and Germany were among the main importers of psittacine birds between 1978 and 2003. The main hypothesis regarding such high volumes of importation to these two destinations is the pet trade, as Psittaciformes is the preferred group in the exotic pet trade [36]. The large number of pet psittacine birds increases the demand for specialized veterinary care. In this regard, universities play an important role in the sanitary evaluation of these introduced psittacine birds. Germany and the United States, similarly to Australia, invest significantly in higher education [34,37]. Therefore, even though these countries do not have large populations of wild or endemic psittacine birds, they contribute the most to the scientific examination of captive birds.

## 5. Most Important Viruses for Psittacine Birds

Most studies (89/118; 75.4%) focused on just one virus. However, 24.6% of the articles showed the analyses of two or more viruses. The virus identified in most articles evaluated herein was the *Circovirus* (62/118), followed by the polyomavirus (26/118), *Bornavirus* (23/118), herpesvirus (20/118), adenovirus (10/118), paramyxovirus (9/118), and *Influenza* (8/118). In addition, eight other viruses were investigated, albeit less frequently in the articles selected: coronavirus, endornavirus, *Flavivirus*, papillomavirus, *Picobirnavirus*, picornavirus, poxvirus, and reovirus. 

*Circovirus*, a genus in the family Circoviridae, is the virus present in most studies. Circoviridae encompasses non-enveloped DNA viruses with a circular genome that is single-stranded, non-segmented, and has 1.7 to 2.1 kb, whose hosts are birds (mainly psittacine birds) and mammals (more frequently swine) [38,39,40,41]. The species that affects psittacine birds is known as the beak and feather disease virus (BFDV). This name is due to the disease it causes, the main clinical signs of which are the gradual loss of feathers or abnormal feathering and an overgrown or irregular beak. BFDV transmission commonly occurs through inhalation or ingestion of the virus, but it can also occur vertically, passing from mother to offspring [42]. This disease causes high morbidity and mortality, especially of chicks. However, subclinical infection can be found in adults [43]. 

The beak and feather disease (BFD) was first identified in Australia in the 1970s [44]. Using phylogeographic analysis, Harkins et al. [45] revealed that the virus was initially introduced in Europe and South Africa and, more recently, in the past 20 years in China and the United States. The data obtained herein corroborate this route of spread of the virus as indicated in the aforementioned study since the identified regions showed up among the countries with the highest scientific production for the diagnosis of the virus. 

## 6. Differential Aspects of Viral Diagnosis in Psittacine Birds 

### 6.1. Methods of Nucleic Acid Extraction

**Figure 4:**
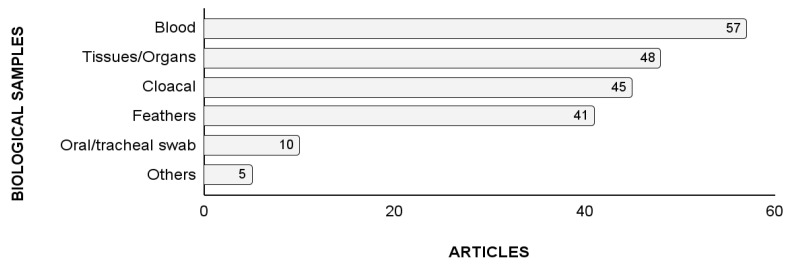
Biological material used for the diagnosis of viruses in parrots according to the number of articles published between 1975 and 2022 and indexed in the Dimensions^®^, PubMed^®^, Scopus^®^, and Web of Science^®^ databases.

It was possible to identify the biological material in 110 out of 118 articles. Studies used one or more biological materials. Blood was the most frequently sampled material, showing up in 57 articles (Figure 4).

For 74 of 118 (62.7%) articles, it was possible to characterize the method used for the extraction of nucleic acids. In a total of 63 articles, the nucleic acid extraction was carried out using only a commercial kit. In nine articles, protocols with different reagents were used: ammonium acetate (used in three articles), sodium acetate, Chelex^®^ 100, DNAzol^®^, phenol/chloroform, sodium iodide, and guanidine thiocyanate. The other two used both kits and conventional protocols (Chelex^®^ 100, DNAzol^®^, and ammonium acetate). 

The commercial kits are extremely precise in the extraction and purification of nucleic acids; thus, it was not a surprise that they were the main extraction method employed. The conventional extraction methods demand higher skills from the researcher in the standardization of protocols, and this might be the reason why they are less commonly used now, taking into account the number of commercial kits available. However, for wild animal screening centers with large groups of birds where there is a need for routine evaluations of several pathogens, it is worthwhile to consider the use of conventional methods for the extraction of nucleic acids in order to reduce the final costs of the polymerase chain reaction (PCR) without interfering in the quality of the genetic material extracted [46,47]. Cost reduction is especially important in countries with a high number of animals received by wild animal screening centers and with low investment in the research area, such as Brazil and Indonesia. Utilization of such conventional methods makes it possible to develop research in these regions, thus bridging the gap regarding viral circulation on a worldwide scale to better predict possible mutations, prevent outbreaks, and define pathogens for each region of the world.

### 6.2. Diagnostic Techniques

Almost 80% (94 of 118) of the studies used PCR as a diagnostic method. We also observed that 74% of the articles (87 of 118) used one or more diagnostic methods in addition to PCR. Immunodiagnostic and sequencing techniques were present in 38% (45 of 118) and 36% (42 of 118) of the articles, respectively. Some articles used cellular and histological evaluation (histopathologic examination and electron microscopy of virus isolation) as auxiliary diagnostics methods, which were present in 26% (31 of 118) of the studies. Other molecular biology techniques used for diagnosis in seven (7 of 118; 6%) of the evaluated articles were: cloning, LAMP (loop-mediated isothermal amplification), Southern blot, and microarray. 

The higher predominance of PCR in the studies is due to its higher sensitivity and specificity compared to other techniques [48]. Sequencing is not always necessary or required for viral diagnosis due to its high cost; however, this technique is essential for the identification of virus variants and phylogenetic studies [49,50,51,52]. On the other hand, immunodiagnostic tests are not recommended for definitive diagnosis, as they are based on the antigen–antibody relationship and dependent on the advancement of infection to obtain a positive diagnosis. However, these tests are very useful as complementary methods to be implemented alongside PCR since this technique also has some limitations. For example, it may generate false-negative results when the viral load is very low, and it may fail to detect the target fragment in the presence of mutations that alter the sequence of the molecular marker, thereby hindering the proper annealing of primers [53,54] 

When the object of study is the virus, several aspects must be taken into consideration for the choice of the diagnostic test. Some viruses progress quickly in the host and cause the bird to die within a few days [55], and in these cases, the quicker the diagnosis, the faster the animal will receive adequate treatment. In this respect, PCR can detect infections in their early stages [48], even prior to the onset of clinical signs, since it depends solely on the presence of the pathogen genome and not on the immune response of the animal. On the other hand, some viruses may not cause any clinical signs [56] and may pose a risk to other species that come in contact with the infected psittacid bird. 

The isolation of the virus in chicken embryo fibroblast culture was commonly used at the beginning of viral research [15,16,57,58], and even though this is a robust method using the direct visualization of the virus, it is a complex, costly, and time-consuming process. The culture is prepared from an 8–10-day-old embryo [15,16], which, after inoculation, is incubated for some hours or days before evaluation using electron microscopy [15,57]. Over the years, this practice has been less widely used for viral diagnosis in psittacid birds, most likely because PCR can be as good a qualitative diagnostic method and is also faster and easier.

### 6.3. Detection of Circovirus: Protocol by Ypelaar et al. [59]

The most used diagnostic method for the detection of Circovirus, the virus more frequently investigated in the articles evaluated herein, was PCR using the primer pair P2 (5′-AACCCTACAGACGGCGAG-3′) and P4 (5′-GTCACAGTCCTCCTTGTACC-3′) designed by Ypelaar et al. [59].

Out of the 62 articles that carried out diagnosis of this virus, 12 did not use primers or did not specify the primer pair used. Out of the 50 remaining articles, 28 (56%) used the primer pair by Ypelaar et al. [59] cited herein, and the other articles (22/50; 44%) used several other less-common primers for their PCR reactions. 

**Figure 5:**
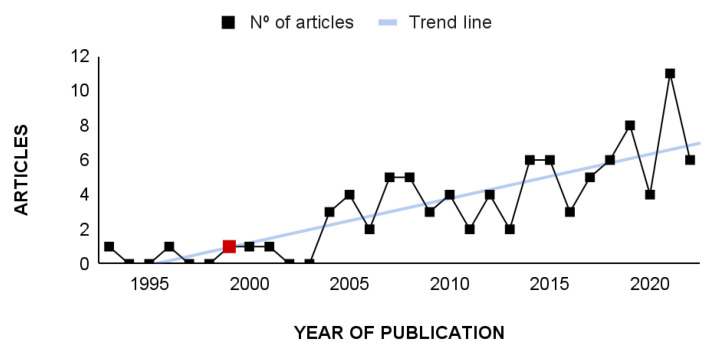
Studies published using PCR as a diagnostic method for viruses in parrots between 1993 and 2022. The red square indicates the work published by Ypelaar et al. [59], in which they described the PCR most used to diagnose Circovirus in parrots.

The article by Ypelaar et al. [59], entitled “A universal polymerase chain reaction for the detection of psittacine beak and feather disease virus”, according to Google Scholar, has been cited in 163 scientific articles, is the basis for several studies, and is considered the gold standard in the diagnosis of BFDV against which new diagnostic techniques are tested for certification [60,61]. Aiming to determine if one PCR reaction could be used to detect all isolates of BFDV reported in Australia, Ypelaar et al. [59] designed seven primer pairs based on several sequences of the genome of the BFDV and tested new pair combinations of “forward” and “reverse” primers for the diagnosis using conventional PCR. The result obtained was the generation of amplicons consistent only for the primers designed based on sequences within the ORF1, which are P2 and P4, and since then, other studies have begun to use this primer pair for the diagnosis of circovírus in birds [62,63,64]. Although it was not the first study to use PCR for viral diagnosis in psittacid birds [9,65], the article by Ypelaar et al. [59] was important for the establishment of PCR, not only for the diagnosis of Circovirus but also in the general diagnosis of viruses in psittacid birds (Figure 5).

## 7. Main Investigated Virus Targets in Psittaciformes

### 7.1. Taxonomic Classification of the Evaluated Birds 

The birds evaluated for the presence of viruses belonged to different families within the order Psittaciformes. Each article had one or more families as the object of study. Psittacidae was the most frequent family in the studies, present in 74/118 (63%) of the articles. Psittaculidae was the second most frequent family, present in 65/118 (55%) of the articles. Cacatuidae ranked close to the first two, included in 62/118 (53%) of the studies. Nestoridae appeared in four (3%) of the studies. Strigopidae was the second least frequent family, present in only one of the evaluated articles. Species from the Psittrichasidae family were not included in any of the studies. Four of the articles included in this review did not identify the evaluated animals at the species level but used common names of birds that could encompass any species within the Psittaciformes, like “parrot” and “psittacine bird”; therefore, it was not possible to identify the families in the aforementioned studies. 

Psittacidae and Psittaculidae are the most diverse families within Psittaciformes. These two families comprise over 90% of the species in the order [1]. The number of studies within each family should be taken into account. Cacatuidae is at least eight times less diverse than Psittacidae and Psittaculidae [1]; however, the number of studies that included birds in these three families was very similar. 

Cacatuidae encompasses species that are highly popular in the pet trade and very sensitive to viral infections, with several reports of severe diseases [64,66,67], including one of the first reports of BFD [44]. It should be emphasized that these birds are mainly distributed in Australia [1], where most renowned researchers in the area of bird virology are located, making the development of research on these birds more possible. 

### 7.2. Allocation of the Evaluated Birds 

Most articles identified the psittacid birds studied as captive-bred (65%; 77/118). Despite this, eight (7%) articles reported that they studied only wild birds, whereas ten (9%) indicated including both captive-bred and wild birds in their study. In 23 (20%) of the selected articles, it was not possible to identify the animal’s allocation. Captivity may cause stress to the bird, which decreases its immunity and thus makes it more susceptible to pathogens. Therefore, it was not unexpected that most studies on viral diagnosis have been carried out on captive-bred birds. Additionally, the capture and immobilization of a wild animal for biological material collection is much more complex than the same procedure with an animal placed in a cage or raised in an avian nursery. Also, as already noted, two among the three main countries of origin of the studies (Germany and the USA) (i) do not have naturally occurring psittacid birds, which makes the study of wild animals inviable in those locations; and (ii) are among the main importers of psittacid birds for the pet market [35], thus increasing the demand for preventive veterinary medicine. 

### 7.3. Findings of Psittacid Birds with Negative Diagnosis for Several Viruses 

Five (5/118; 4%) of the articles obtained negative results for the viral diagnosis of all tested psittacid birds. Of these five, three articles targeted individuals in the family Psittacidae, and the only country with more than one article with negative findings was Brazil. Despite these findings, the evidence found in this review is not sufficient to state that there is no viral circulation among psittacid birds in Brazil, nor that the Psittacidae birds are more resistant to viral infections than the remaining members of this order (Table 5).

## 8. Conclusions and Future Directions

Viral infections have been diagnosed in five families within the Psittaciformes (Psittacidae, Psittaculidae, Cacatuidae, Nestoridae, and Strigopidae) and are found in all continents except Antarctica. The countries that contribute the most to the study of viral diagnosis are the United States, Australia, and Germany, and their most renowned researchers are associated with public universities. An average of 2.5 articles on the diagnosis of viruses in psittacid birds were published per year over the 47 years of study evaluated (1975–2022), and 2021 holds the highest productivity. Blood is the most frequently sampled material, and commercial kits are the most commonly used means of extracting DNA. The main methods used for viral diagnosis are based on molecular techniques such as PCR. Circovirus is the most widely distributed and most frequently diagnosed virus in psittacid birds. The most used diagnostic method for the detection of Circovirus is PCR, using the primer pair P2/P4 designed by Ypelaar et al. [59]. Three other viruses of interest in psittacid birds were highlighted in the articles evaluated: polyomavirus, *Bornavirus*, and herpesvirus. Most studies investigated the presence of viruses in captive-bred psittacid birds. 

We strongly recommend the inclusion of viral diagnosis in protocols of epidemiological monitoring of captive-bred psittacid birds. Taking into account the wide distribution and the possibility of subclinical infections [43], viruses should be considered focal pathogens for those birds released into wild programs. The reintroduction of a pathogen-infected animal into the wild may decrease the long-term survival chances of introduced animals and may cause sickness or death of wild animals more susceptible to infections, potentially affecting meat-producing livestock animals as well as humans [73,74,75]. Moreover, the higher the viral transmission, the higher the mutation rate, which can generate more aggressive or more transmissible virus variants [76].

Further research on viruses in psittacid birds in countries that harbor a large diversity of these birds, such as those in Latin America, should be encouraged. For this purpose, besides government financial incentives, more programs should be developed to make the diagnostic methods more accessible and facilitate a widespread viral investigation not only in captive-bred but also in wild birds. The evaluation of wild birds would make it possible to: (i) increase knowledge on the infection and dissemination dynamics of these pathogens, (ii) discover new viruses or variants, and (iii) predict possible mutations that could lead to host jumps to other animal species including other novel viruses of zoonotic origin to humans.

## Figures and Tables

**Table 1:** Search descriptors used to find articles on virus diagnosis in psittacine birds (Psittaciformes) in four databases.

Database	Search Descriptors
Dimensions^®^	(virus AND (parrot OR Psittaciformes OR Psittacidae OR psittacine OR Psittacid) AND (Diagnosis OR Detection)) → Search in: Title and abstract
PubMed^®^	(“virus”) AND (“parrot” OR “Psittaciformes” OR “Psittacidae” OR “psittacine” OR “Psittacid”) AND (“Diagnosis” OR “Detection”)
Scopus^®^	(AUTHKEY (“virus”) OR TITLE (“virus”) OR ABS (“virus”)) AND ((AUTHKEY ((“parrot” OR “Psittaciformes” OR “Psittacidae” OR “psittacine” OR “Psittacid”) AND (“Diagnosis” OR “Detection”)) OR TITLE (((“parrot” OR “Psittaciformes” OR “Psittacidae” OR “psittacine” OR “Psittacid”)) AND (“Diagnosis” OR “Detection”)) OR ABS ((“parrot” OR “Psittaciformes” OR “Psittacidae” OR “psittacine” OR “Psittacid”) AND (“Diagnosis” OR “Detection”))))
Web of Science^®^	(((TI = (virus)) OR AK = (virus)) OR AB = (virus)) AND (((((((TI = (parrot)) OR TI = (Psittaciformes)) OR TI = (Psittacidae)) OR TI = (psittacine)) OR TI = (Psittacid)) AND ((TI = (Diagnosis)) OR TI = (Detection))) OR ((((((AB = (parrot)) OR AB = (Psittaciformes)) OR AB = (Psittacidae)) OR AB = (psittacine)) OR AB = (Psittacid)) AND ((AB = (Diagnosis)) OR AB = (Detection))) OR ((((((AK = (parrot)) OR AK = (Psittaciformes)) OR AK = (Psittacidae)) OR AK = (psittacine)) OR AK = (Psittacid)) AND ((AK = (Diagnosis)) OR AK = (Detection))))

**Table 2:** Articles recovered, discarded (after evaluation and exclusion following the established criteria), and selected from a search regarding viral diagnosis in psittacine birds in four databases.

Database	N° of Articles Recovered	N° of Articles Discarded (%)	N° of Articles Selected	N° of Articles Selected and Exclusive (Except for Dimensions^®^)
Dimensions^®^	135	38 (28.1%)	97	-
PubMed^®^	121	33 (27.3%)	88	13
Scopus^®^	115	31 (27.0%)	84	6
Web of Science^®^	98	28 (28.6%)	70	0
N° of selected articles that did not show up at Dimensions^®^ but were repeated in more than one of the other three databases				2
Total number of articles selected that did not show up at Dimensions^®^				21

**Table 3:** The five authors and countries with the greatest impact on bibliographic production on virus detection in parrots between 1975 and 2022. Data generated by the VOSViewer^®^ version 1.6.18 software from articles indexed in the Dimensions^®^ database.

Bibliographic Production	Documents	Citations	Total Link Strength
Author			
Raidal, S. R.	10	390	45
Lierz, M.	6	168	49
Kaleta, E. F.	5	102	33
Phalen, D. N.	5	83	15
Bonne, N.	4	135	17
Countrie (organization)			
United States	17	574	6
Australia	17	490	3
Germany	15	306	4
South Africa	4	70	6
Brazil	4	19	0

Source: VOSviewer^®^ version 1.6.18. Selection/classification: 1. Documents, 2. Citations, 3. Total link strength.

**Table 4:** The 15 organizations with the greatest impact on bibliographic production on virus detection in parrots between 1975–2022. Data generated by the VOSViewer^®^ version 1.6.18 software from articles indexed in the Dimensions^®^ database.

Organization	Country	Funding	D *	C *	T *
Texas A&M University	USA	Public	8	151	2
Murdoch University	Australia	Public	7	253	7
Charles Sturt University	Australia	Public	7	173	8
University of Giessen	Germany	Public	7	146	12
Leipzig University	Germany	Public	5	105	13
Ludwig-Maximilians-Universität München	Germany	Public	4	85	6
University of Cape Town	África do Sul	Public	4	70	9
China Agricultural University	China	Public	4	4	2
University of Freiburg	Germany	Public	3	65	10
Deakin University	Australia	Public	3	29	3
Friedrich-Loeffler-Institut	Germany	Public	3	18	10
USP—Universidade de São Paulo	Brazil	Public	3	12	4
University of California, San Francisco	USA	Public	2	288	2
Columbia University	USA	Private	2	100	2
Freie Universität Berlin	Germany	Public	2	77	1

* D = documents; C = citations; T = total link strength. Source: VOSviewer^®^ version 1.6.18. Selection/classification: 1. Documents, 2. Citations, 3. Total link strength.

**Table 5:** Articles on diagnosis of viruses in parrots with only negative results in the tests performed.

Author	C.O.B *	Diagnostic Methods	Family	Allocation	Virus Investigated
Buyse et al. [68]	South Africa	PCR	Psittacidae	Wild	*Circovirus*
Bitrus et al. [69]	Nigeria	qRT-PCR	Psittacine bird **	Captive	*Coronavirus*
Murray et al. [70]	USA	Histopathology, immunohistochemistry and RT-PCR	Cacatuidae	Captive	*Bornavirus*
Vaz et al. [71]	Brazil	Multiplex RT-PCR	Psittacidae	Wild	*Flavivirus*, *Influenza* and *Paramyxovirus*
Saidenberg et al. [72]	Brazil	PCR and RT-PCR	Psittacidae	Captive and wild	*Coronavirus*, *Herpesvirus*, *Influenza*, *Paramyxovirus* and *Poxvirus*

* Country of origin of birds. ** Family not identified in the article.

## Data Availability

Not applicable.
